# Supplementary material for: Barrier-free reverse-intersystem crossing in organic molecules by strong light-matter coupling
Source: Nat Commun. 2021 May 31;12:3255. doi: 10.1038/s41467-021-23481-6 (PMC8167092; doi:10.1038/s41467-021-23481-6)
Supplement: Supplementary file 1 — Supplementary Information [file 41467_2021_23481_MOESM1_ESM.pdf]

Supplementary Information

Barrier-free reverse-intersystem crossing in organic molecules by strong light-matter coupling

Yi Yu[1,2], Suman Mallick[1,2], Mao Wang[1] and Karl Börjesson[1*]

[1] Department of Chemistry and Molecular Biology, University of Gothenburg, Kemigården 4, 412 96 Gothenburg, Sweden.
[2] These authors contributed equally: Yi Yu, Suman Mallick.
Corresponding Authors: E-mail: karl.borjesson@gu.se (K.B.)

Contents

1. Supplementary Note 1 - Synthetic Procedures and Analytical Data 2

2. Supplementary Figures 10

3. Supplementary Tables 20

4. Supplementary References 22

1. Supplementary Note 1 – Synthetic Procedures and Analytical Data

All starting materials were purchased from Sigma-Aldrich Chemical Co. and used without further purification. All moisture- and oxygen-sensitive reactions were carried out using Schlenk techniques in oven-dried glassware. Solvents used for moisture and oxygen-sensitive reactions were dried using an MBraun MB SPS-800 solvent purification system and, if necessary, degassed by freeze-pump-thaw cycles and stored over 4-Å molecular sieves under argon atmosphere. Flash chromatography was performed using a Teledyne CombiFlash EZ prep and normal-phase silica. 1H NMR (nuclear magnetic resonance) spectra were recorded on a Varian spectrometer at 400 MHz, J-coupling values are given in hertz, and chemical shifts are given in parts per million using tetramethysilane, with 0.00 ppm as an internal standard.

Synthesis of Compounds 3DPyM-pDTC

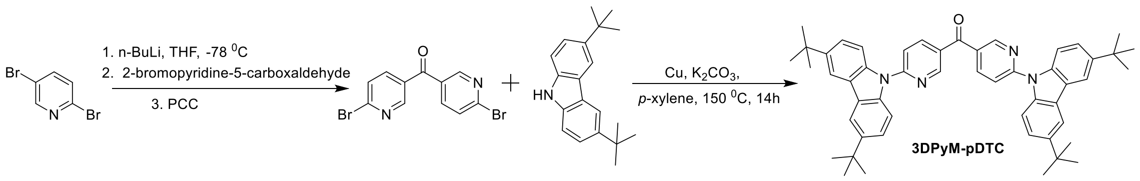

Supplementary Scheme 1 Synthesis of 3DPyM-pDTC

3DPyM-pDTC was synthesized following a literature reported procedure with some modifications.[1]

Synthesis of bis(6-bromopyridin-3-yl) methanone: To a stirred solution of 2,5-dibromopyridine (2.0 g, 8.43 mmol) in dry THF (35 mL) at -78 °C was added n-BuLi (2.5 M) (3.4 mL, 8.43 mmol) and stirred for 1 h at the same temperature. With this solution, 2-bromopyridine-5-carboxaldehyde (1.57 g, 8.43 mmol) in THF (8 mL) was added dropwise and the reaction mixture was allowed to stir for another 2 h at -78 °C. The mixture was quenched with aqueous HCl (1 M) at 0 °C and partitioned between water and DCM (50 ml). The organic layer was washed with water (20 ml) two times and dried over Na_2SO_4. To this DCM layer, PCC (pyridinium chlorochromate) (3.63 g, 16.87 mmol) was added and stirred at room temperature overnight. The reaction mixture was then filtered through a Celite pad and washed with DCM. Evaporation of solvent followed by column chromatography purification gave compound **A**, 1.148g (39% yield). The compound was characterized by ^{1}H NMR, which matched well with the literature values.[1]

Synthesis of 3DPyM-pDTC: In a flame dried sealed tube (bis(6-bromopyridin-3-yl)methanone) (0.5 g, 1.45 mmol), 3,6-di-tert-butyl-9H-carbazole (0.81 g, 2.9 mmol), Cu (0.19 g, 2.9 mmol), and K_2CO_3 (0.82 g, 5.85 mmol) were added. The tube was then sealed then evacuated and purged with Argon three times. Dry *p*-xylene (4 mL) was added under Argon atmosphere and the reaction was stirred at 150 °C for 12 h. After that, the mixture was diluted with ethyl acetate (20 mL) and filtered through a Celite pad, which was washed two times with 20 mL of ethyl acetate. The combined filtrate was concentrated, followed by column chromatography purification using *n*-hexane/EtOAc as the eluent to give 3DPyM-pDTC as a greenish yellow solid (472 mg, 43% yield), characterized through ^{1}H NMR.

^{1}H NMR (400 MHz, Chloroform-*d*), δ(ppm): 9.20 (d, 2 H), 8.43 (dd, *J* = 8.4 Hz, J = 2.4 Hz, 2 H), 8.12 (s, 4 H), 8.03 (d, *J* = 8.8 Hz, 4 H), 7.87 (d, *J* = 8.4 Hz, 2 H), 7.54 (dd, *J* = 8.8 Hz, 4 H), 1.48 (s, 36 H);

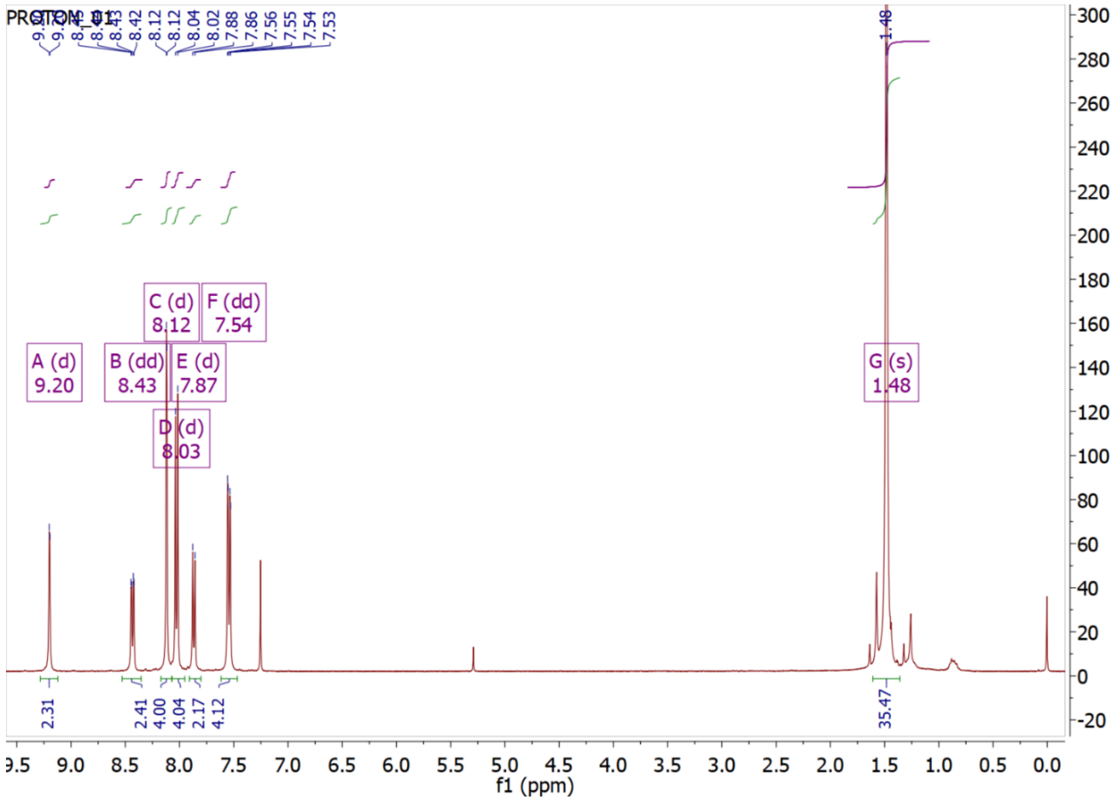

Supplementary NMR 1 - ^{1}H NMR (400 MHz, Chloroform-*d*) of 3DPyM-pDTC

Synthesis of Boron difluoride curcuminoid derivative

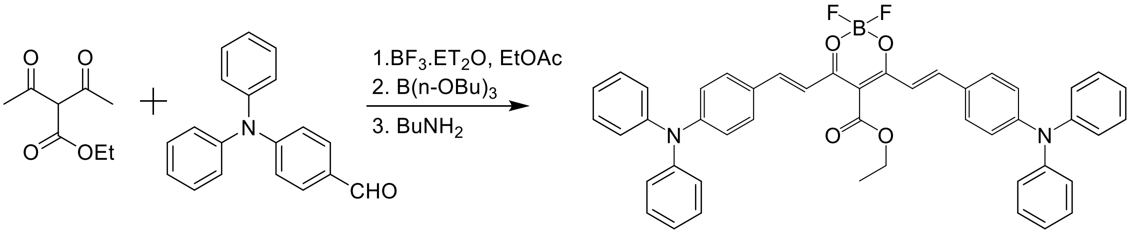

Boron difluoride curcuminoid derivative

Supplementary Scheme 2 Synthesis of Boron difluoride curcuminoid derivative

The boron difluoride curcuminoid derivative was synthesized following a literature reported procedure with some modifications.[2]

In a 50 mL flask, ethyl diacetoacetate (228 µL, 1.463 mmol, 1 eq) and BF_3 Et_2O (199 µL, 1.609 mmol, 1.1 eq) in 3 mL ethyl acetate was heated for 30 min at 50 ºC in air. 4-(*N*,*N*-diphenylamino)-benzaldehyde (1 g, 3.658 mmol, 2.5 eq) and $B(n\text{-}OBu)_3$ (0.987 mL, 3.658 mmol, 2.5 eq) was dissolved into 12 mL ethyl acetate, and the solution was injected into the first mixture. The reaction was kept at 50-60 ºC for another 30 min. Then, a first portion of $BuNH_2$ (145µL, 1.45mmol, 1eq) was added dropwise into the reaction. After 6 h, a second portion of $BuNH_2$ (145 µL, 1.45 mmol, 1 eq) was added, and the reaction was kept at 50 ºC overnight. All the solvents were evaporated, and the crude product was obtained by flash column chromatography on silica, using DCM. Further purification was done by multiple precipitations in DCM/pentane mixtures, giving a shiny dark green powder (522 mg, 48% yield). ^{1}H NMR matched well with reported literature.[2]

^{1}H NMR (400 MHz, Chloroform-*d*), δ(ppm): 8.10 (d, *J*= 15.1 Hz, 2H), 7.45 (d, *J*= 8.8 Hz, 4H), 7.33 (m, 8H), 7.15 (m, 14H), 6.97 (d, *J*= 8.8 Hz, 4H), 4.40 (q, 2H), 1.42 (t, 3H);

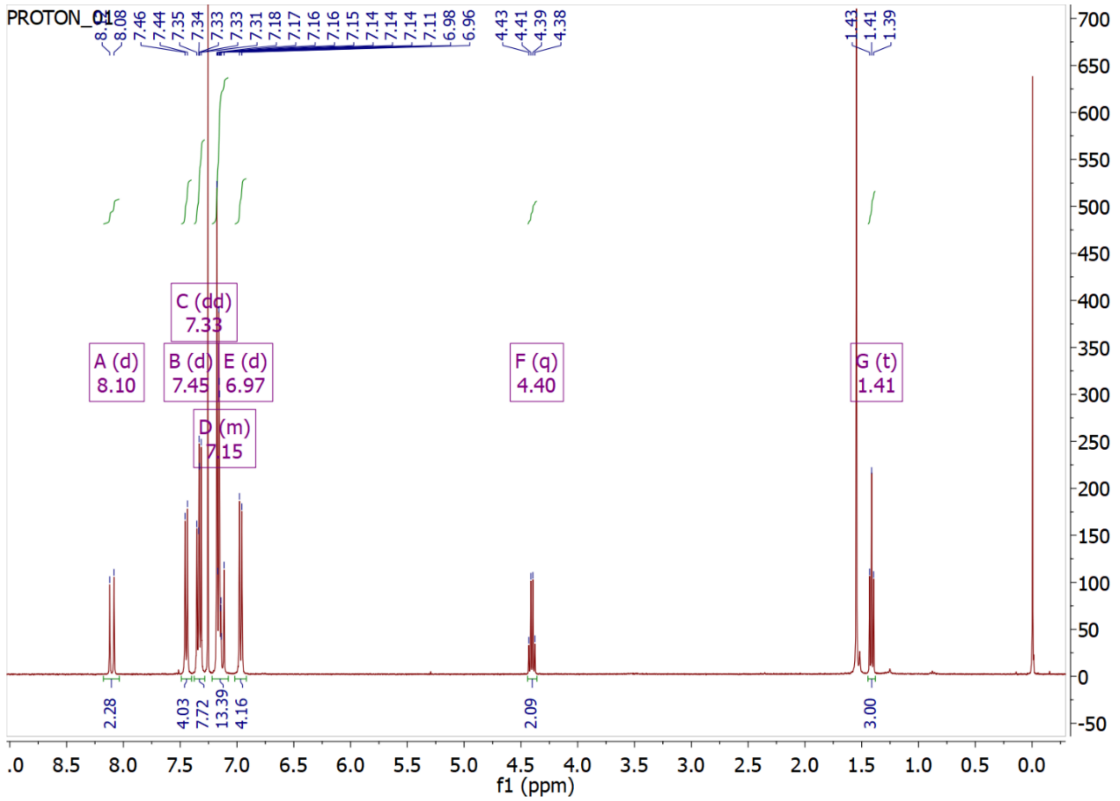

Supplementary NMR 2 - ^{1}H NMR (400 MHz, Chloroform-*d*) of boron difluoride curcuminoid derivative

Synthesis of DABNA-1 and DABNA-2

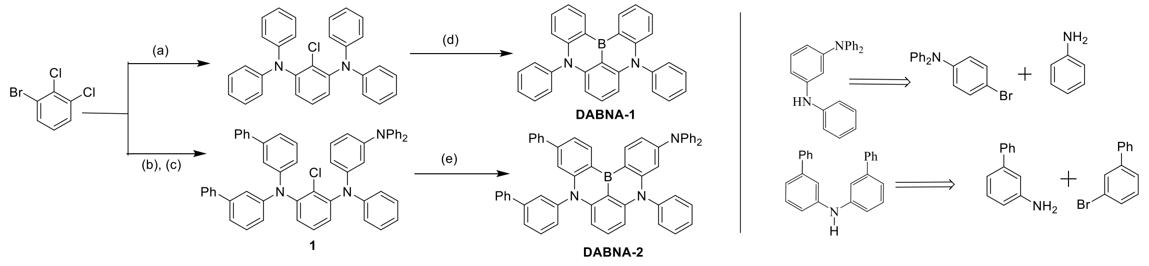

Supplementary Scheme 3 Synthesis of DABNA-1 and DABNA-2

DABNA-1 and DABNA-2 was synthesized following a literature reported procedure with some modifications.[3] Due to the lack of commercial availability of starting materials, compounds used in the steps **b** and **c** was prepared following the similar coupling protocol as mentioned in step **a** for the synthesis of DABNA-1.

Synthesis of N^1,N^1,N^3-triphenylbenzene-1,3-diamine: 3-Bromo-*N*,*N*-diphenylaniline (1.165 g, 5 mmol) and aniline (0.465 g, 5 mmol) were heated to 120 ºC for 24 h in 20 ml *o*-xylene with potassium tert-butylate (0.68g, 6 mmol) as base and Pd(AMPHOS)$_2$Cl$_2$ (0.105 g, 0.15 mmol) as catalyst under Argon atmosphere. The solvent was removed under reduced pressure and the resulting mixture was extracted with dichloromethane and purified through column chromatography to give the title compound as a white solid (1.2 g, 71%).
^{1}H NMR (400 MHz, Chloroform-*d*), δ(ppm): 7.37-7.29 (m, 6H), 7.26-7.20 (m, 5H), 7.14-7.08 (m, 4H), 6.99 (t, 1H), 6.85-6.83 (m, 2H), 6.77-6.75 (dd, 2H), 5.61 (s, 1H, NH proton)

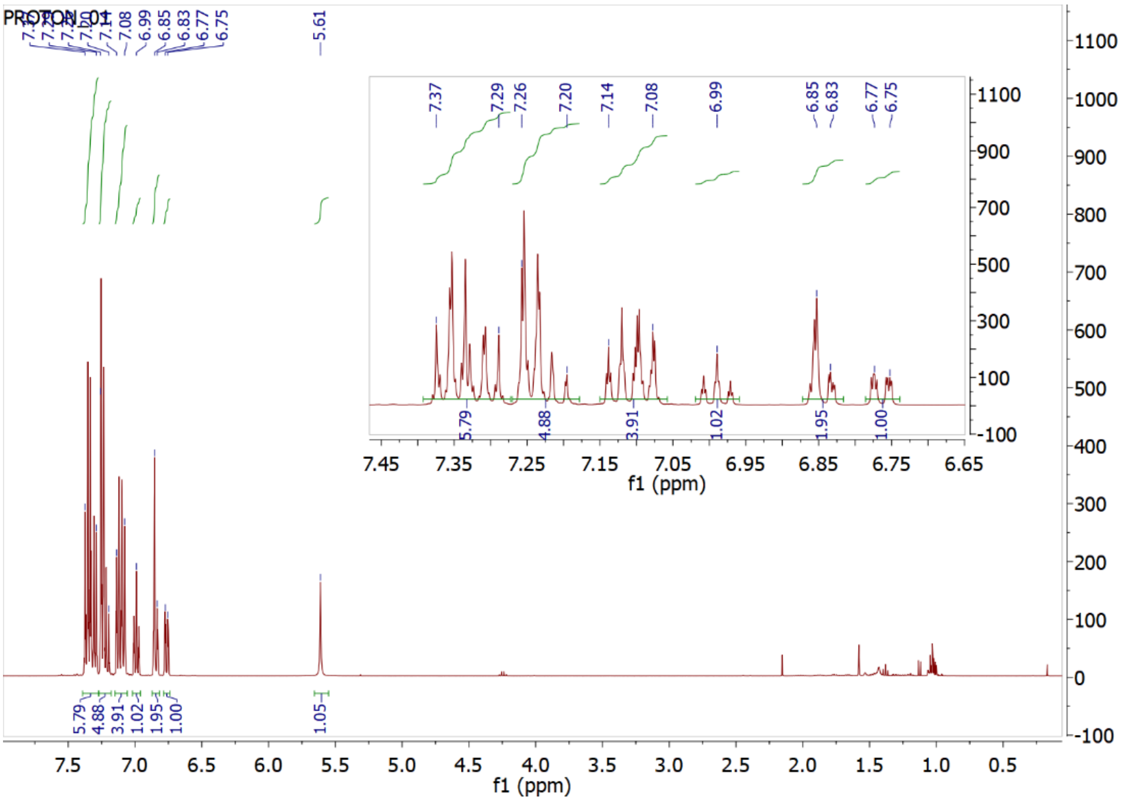

Supplementary NMR 3 - ^{1}H NMR (400 MHz, Chloroform-*d*) of *N*[1],*N*[1],*N*[3]-triphenylbenzene-1,3-diamine

Synthesis of di([1,1'-biphenyl]-3-yl)amine: 3-Bromobiphenyl (1.165 g, 5mmol) and 3-aminobiphenyl (0.846 g, 5 mmol) were heated to 150 °C for 24 h in 20 ml *o*-xylene with potassium tert-butylate (0.68g, 6 mmol) as base and Pd(AMPHOS)$_2$Cl$_2$ (0.105 g, 0.15 mmol) as catalyst under Argon atmosphere. The solvent was removed under reduced pressure and the resulting mixture was extracted with dichloromethane and purified through column chromatography to give the title compound as a white solid (1.22 g, 76%).

^{1}H NMR (400 MHz, Chloroform-*d*), δ(ppm): 7.67-7.65 (m, 4H), 7.50 (m, 4H), 7.42 (m, 6H), 7.27 (m, 2H), 7.19 (dd, 2H), 5.87 (broad, 1H, NH proton)

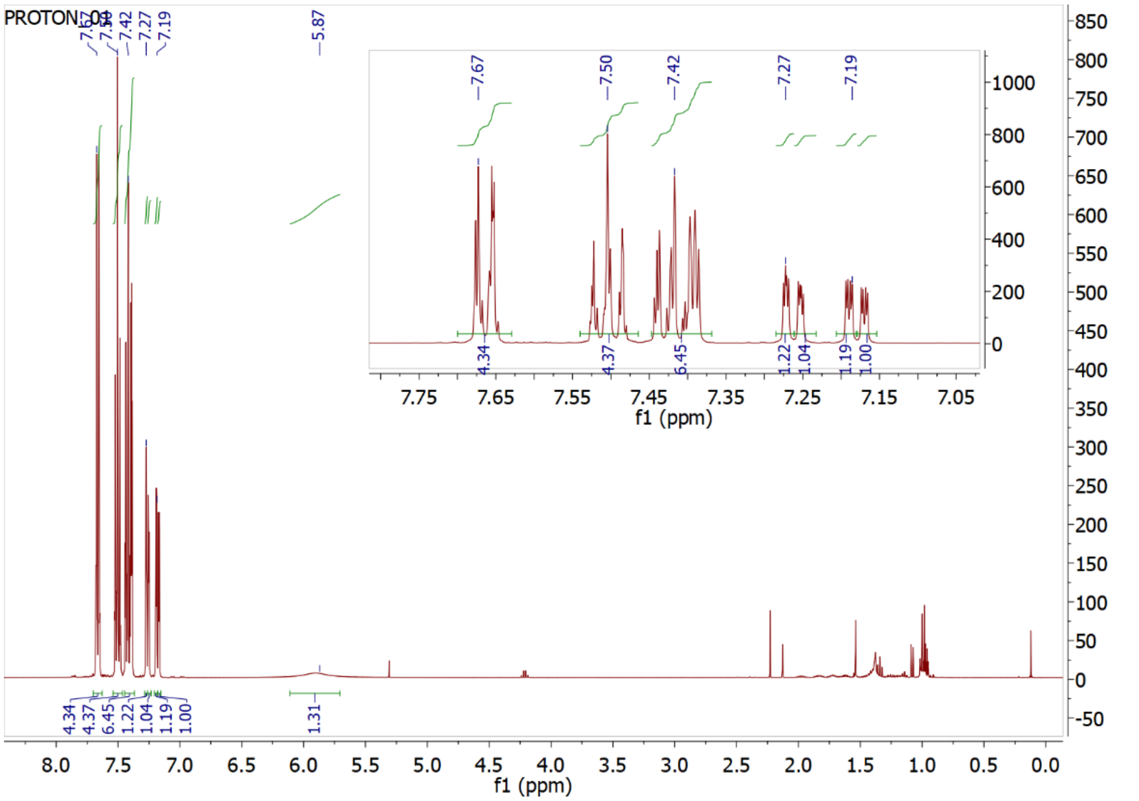

Supplementary NMR 4 - ^{1}H NMR (400 MHz, Chloroform-*d*) of di([1,1'-biphenyl]-3-yl)amine

Synthesis of DABNA-1: Step (a) Diphenylamine (1.5 g, 8.86 mmol), sodium tert-butoxide (0.97 g, 10.15 mmol), Pd(AMPHOS)$_2$Cl$_2$ (30 mg, 0.04 mmol) and 1-bromo-2,3-dichlorobenzene (0.90 g, 4.02 mmol) were dissolved in dry o-xylene (12 mL) under a nitrogen atmosphere. After stirring at 80 ºC for 2 h followed by at 120 ºC for 3 h, the reaction mixture was cooled to room temperature. The solvent was removed under reduced pressure and the resulting mixture was extracted with dichloromethane and purified through column chromatography to get 2-chloro-*N*^{1}, *N*^{1}, *N*^{3}, *N*^{3}-tetraphenylbenzene-1,3-diamine (2.06 g, 52% yield). The compound was characterized by ^{1}H NMR, which matched well with the literature values.[3]

Step (d) A solution of tert-butyllithium in pentane (1.65 mL, 1.60 M, 2.64 mmol) was added slowly to a solution of 2-chloro-*N*^{1}, *N*^{1}, *N*^{3}, *N*^{3}-tetraphenylbenzene-1,3-diamine (1.0 g, 2.2 mmol) in tert-butylbenzene (8 mL) at –30 ºC under a nitrogen atmosphere. After stirring at 60 ºC for 2 h, boron tribromide (0.25 mL, 2.69 mmol) at –30 ºC, the reaction mixture was stirred at room temperature for 0.5 h. *N*,*N*-Diisopropylethylamine (0.78 mL, 4.55 mmol) was then added at 0 ºC and the reaction mixture was allowed to warm to room temperature. After stirring at 120 ºC for 3 h, the reaction mixture was cooled to room temperature. An aqueous solution of sodium acetate 10 ml (13.0 g in 100 mL) and ethyl acetate (30 mL) was added to the reaction mixture and the formed aqueous layer was separated and extracted with ethyl acetate (30 mL). The combined organic layer was condensed in vacuo and purified through column chromatography to give DABNA-1 (264 mg, yield 28%). The compound was characterized by ^{1}H NMR, which matched well with the literature values.[3]

^{1}H NMR (400 MHz, Chloroform-*d*), δ(ppm): 6.15 (d, *J* = 8.5 Hz, 2H), 6.77 (d, *J* = 8.7 Hz, 2H), 7.24–7.29 (m, 3H), 7.38-7.44 (m, 6H), 7.59 (tt, *J* = 1.4, 7.6 Hz, 2H), 7.70 (t, *J* = 7.6 Hz, 4H), 8.96 (dd, *J* = 1.6, 7.8 Hz, 2H)

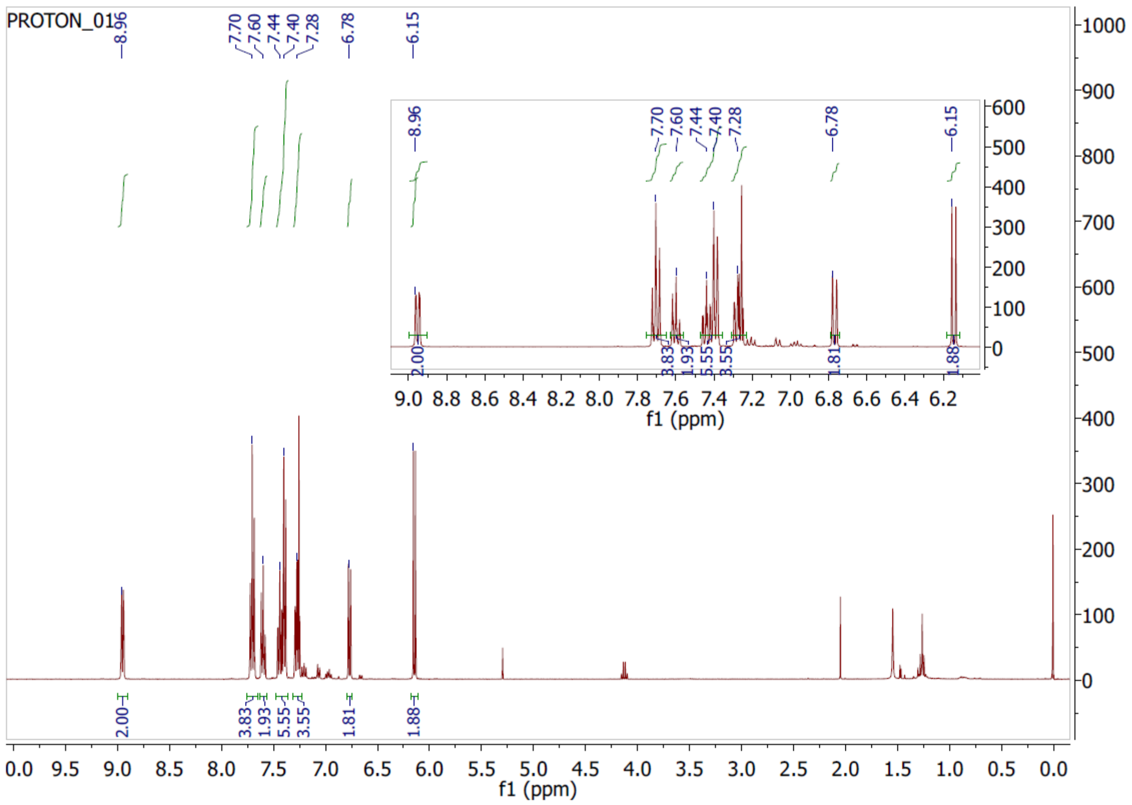

Supplementary NMR 5 - ^{1}H NMR (400 MHz, Chloroform-*d*) of DABNA-1

Synthesis of DABNA-2: Step (b) *N*^{1},*N*^{1},*N*^{3}-Triphenylbenzene-1,3-diamine (1.03 g, 3.08 mmol), sodium tert-butoxide (0.448 g, 4.66 mmol), Pd(AMPHOS)$_2$Cl$_2$ (12 mg, 0.015 mmol) and 1-bromo-2,3-dichlorobenzene (0.7 g, 3.1 mmol) were dissolved in o-xylene (8 mL) under a nitrogen atmosphere. After stirring at 90 ºC for 2.5 h, the reaction mixture was cooled to room temperature. Ethyl acetate (30 mL) and water (5 mL) were added to the reaction mixture and then the organic layer was separated and washed with water twice. The organic layer was condensed in vacuo and purified by silica gel column chromatography to get *N*^{1}-(2,3-dichlorophenyl)-*N*^{1},*N*^{3},*N*^{3}-triphenylbenzene-1,3-diamine (1.13 g, 76% yield). The compound was characterized by ^{1}H NMR, which matched well with the literature values.[3]

Step (c) The product from step **b**, *N*^{1}-(2,3-dichlorophenyl)-*N*^{1},*N*^{3},*N*^{3}-triphenylbenzene-1,3-diamine (1.0 g, 2.08 mmol), was added to a solution of di([1,1'-biphenyl]-3-yl)amine (0.66. g, 2.08 mmol), sodium tert-butoxide (0.30 g, 3.12 mmol) and Pd(AMPHOS)$_2$Cl$_2$ (15 mg, 0.02 mmol) in o-xylene (6 mL) under a nitrogen atmosphere. After stirring at 120 ºC for 1 h, the reaction mixture was cooled to room temperature. Ethyl acetate (30 mL) and water (5 mL) were added and the organic layer was separated and washed with water twice. The combined organic layers were condensed in vacuo and purified by silica gel column chromatography to get the product **1** (1.16 g, 75% yield). The compound was characterized by ^{1}H NMR, which matched well with the literature values.[3]

Step (e) A solution of tert-butyllithium in pentane (1.63 mL, 1.60 M, 2.62 mmol) was added slowly to a solution of **1** (1.0 g, 1.3 mmol) in tert-butylbenzene (10 mL) at 0 ºC under a nitrogen atmosphere. After stirring at 60 ºC for 3 h, boron tribromide (0.65 g, 2.61 mmol) at –50 ºC. The reaction mixture was stirred at room temperature for 0.5 h, then *N*,*N*-diisopropylethylamine (0.33 g, 2.59 mmol) was added at 0 ºC and the reaction mixture was allowed to warm to room temperature again. After stirring at 120 ºC for 1.5 h, the reaction mixture was cooled to room temperature. An aqueous solution of sodium acetate, 5 mL, (13.0 g in 100 mL water with ice) and ethyl acetate (30 mL) was added to the reaction mixture. The organic layer was separated, washed with water twice,

and then condensed in vacuum. Purification by silica gel column chromatography gave DABNA-2 (270 mg, 27% yield). The compound was characterized by ^{1}H NMR, which matched well with the literature values.[3]

^{1}H NMR (400 MHz, Chloroform-*d*), δ(ppm): 6.11 (d, *J* = 8.0 Hz, 1H), 6.19-6.22 (m, 2H), 6.97 (dd, *J* = 2.3, 8.6 Hz, 1H), 7.01–7.04 (m, 3H,), 7.13–7.23 (m, 11H), 7.27-7.50 (m, 13H), 7.65–7.70 (m, 3H), 7.77 (t, *J* = 8.0 Hz, 1H), 7.84 (m,1H), 8.77 (d, *J* = 8.6 Hz, 1H), 8.95 (d, *J* = 8.0 Hz, 1H)

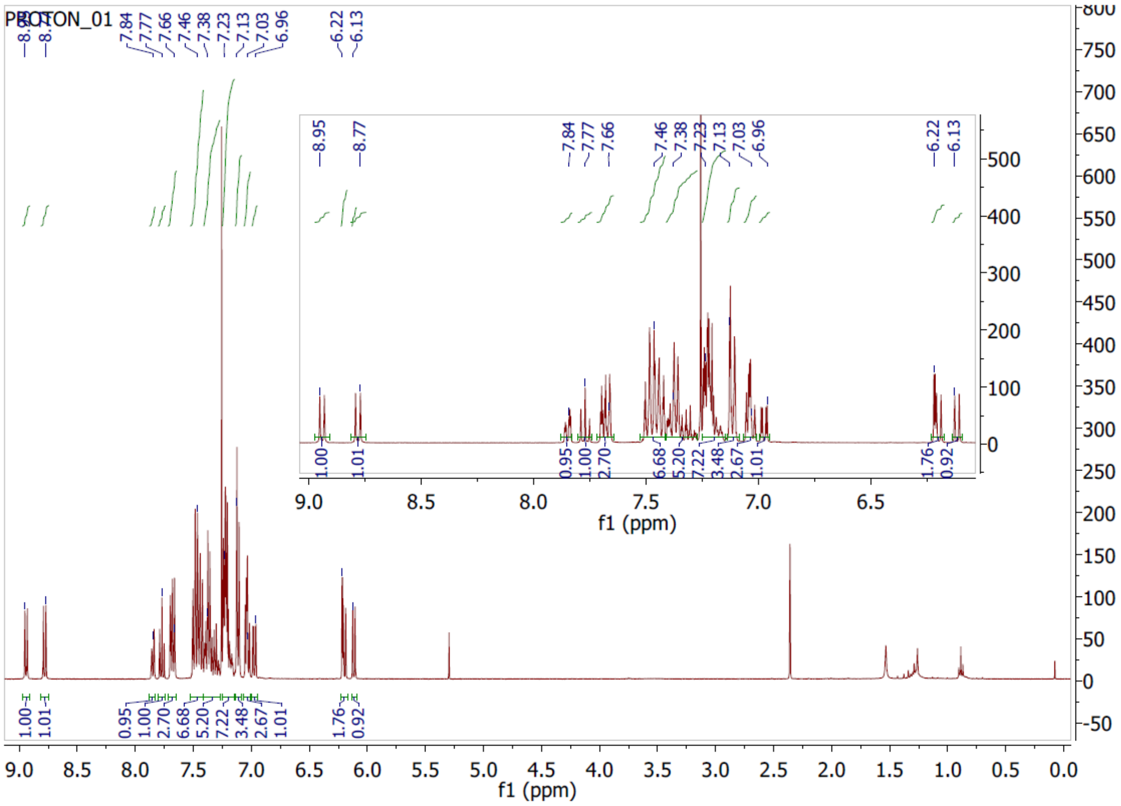

Supplementary NMR 6 - ^{1}H NMR (400 MHz, Chloroform-*d*) of DABNA-2

Synthesis of TBN-TPA

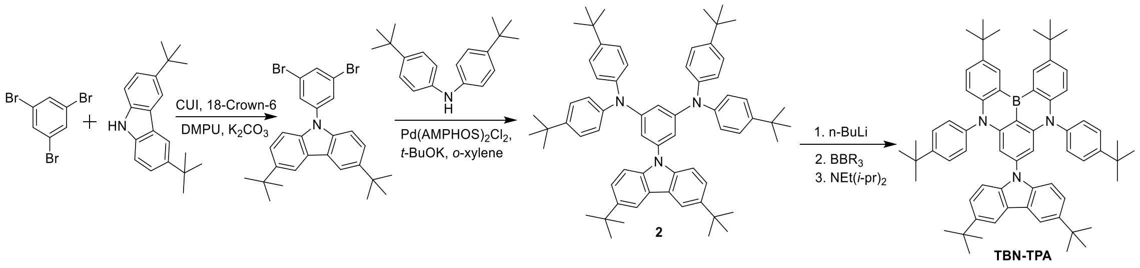

Supplementary Scheme 4 Synthesis of TBN-TPA

Synthesis of 3,6-di-*tert*-butyl-9-(3,5-dibromophenyl)-9H-carbazole: A mixture of 1,3,5-tribromobenzene (1.5 g, 4.7 mmol) and 3,6-di-tert-butyl-9H-carbazole (1.33 g, 4.7 mmol) with CuI (5.71 mg, 0.03 mmol) as catalyst, 18-crown-6 (7.89 mg, 0.03 mmol) as phase transfer catalyst, and potassium carbonate (0.654g, 4.7 mmol) as base, in 3.5 mL 1,3-dimethyl-tetrahydropyrimidin-2(1H)-one (DMPU) were heated to 190 ºC overnight in a sealed tube. The resulting mixture was diluted with 50 ml dichloromethane, washed with 20 ml water, two times, and then dried with Na_2SO_4. Finally, column chromatography gave 3,6-di-tert-butyl-9-(3,5-dibromophenyl)-9H-carbazole (1.23 g, 50% yield). The compound was characterized by ^{1}H NMR, which matched well with the literature values.[4]

Synthesis of 2: Bis(4-(tert-butyl)phenyl)amine (1.40 g, 5mmol) and 3,6-di-*tert*-butyl-9-(3,5-dibromophenyl)-9H-carbazole (1.02 g, 2 mmol) were heated to 150 °C for 24 h in 20 mL dry o-xylene with potassium *tert*-butylate (0.68 g, 6 mmol) as base and Pd(AMPHOS)$_2$Cl$_2$ (42 mg, 0.06 mmol) as catalyst. The solvent was removed under reduced pressure and the resulting mixture was extracted with dichloromethane and purified through column chromatography to get the product **2** (1.21 g, 68% yield). The compound was characterized by ^{1}H NMR, which matched well with the literature values.[4]

Synthesis of TBN-TPA: To a solution of **2** (1 g, 1.09 mmol) in dry *tert*-butylbenzene was added *n*-BuLi (0.52 ml, 2.5 M) dropwise at 0 °C. The reaction mixture was stirred for 20 minutes before heated to 90 °C and stirred for an additional 4 hours. Then 0.14 mL boron tribromide (1.5 mmol) was added carefully at -42 °C through a syringe and kept stirring for additional 30 min. The reaction was slowly warmed to room temperature and kept stirring overnight. The resulting solution was cooled in an ice bath and then 0.33 ml (2 mmol) *N*-ethyldiisopropylamine was added slowly. The reaction was heated to 100 °C and left for react for 24 hours. After the reaction cooled down to room temperature, the reaction mixture was carefully quenched by addition of iced-water. The mixture was filtered through a celite pad, washed with toluene (50 mL) and the solvent was removed under vacuum. The residue was purified through column chromatography with toluene as eluent to yield TBN-TPA as a light yellowish-green solid (0.284 g, 26% yield). The compound was characterized by ^{1}H NMR, which matched well with the literature values.[4]

^{1}H NMR (400 MHz, Chloroform-*d*), δ(ppm): 9.09 (d, *J* = 18.9, 2.1 Hz, 2H), 8.38 (d, *J* = 1.8 Hz, 1H), 8.21 (d, *J* = 2.0 Hz, 1H), 7.57 – 7.41 (m, 4H), 7.38 – 7.15 (m, 11H), 7.03 (d, *J* = 9.0 Hz, 1H), 6.69 (d, *J* = 9.0 Hz, 1H), 6.22 (m, 1H), 1.68-1.30 (m, 54H)

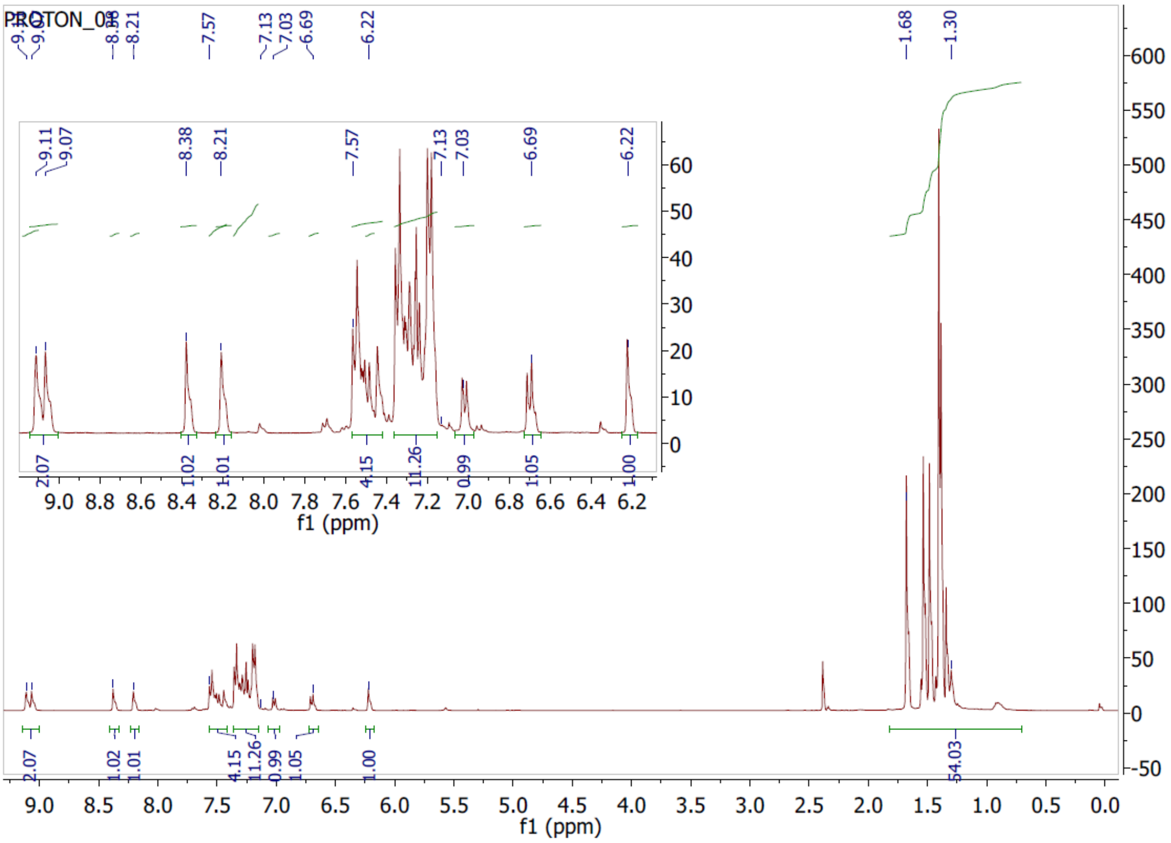

Supplementary NMR 7 - ^{1}H NMR (400 MHz, Chloroform-*d*) of TBN-TPA

2. Supplementary Figures

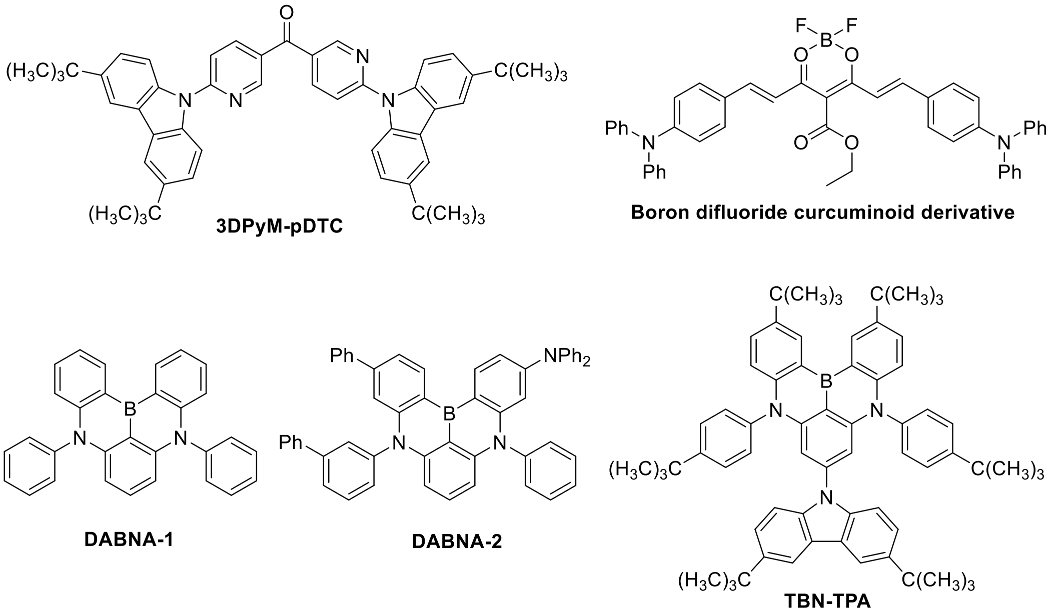

Supplementary Figure 1 Structures of the synthesized molecules used in this study.

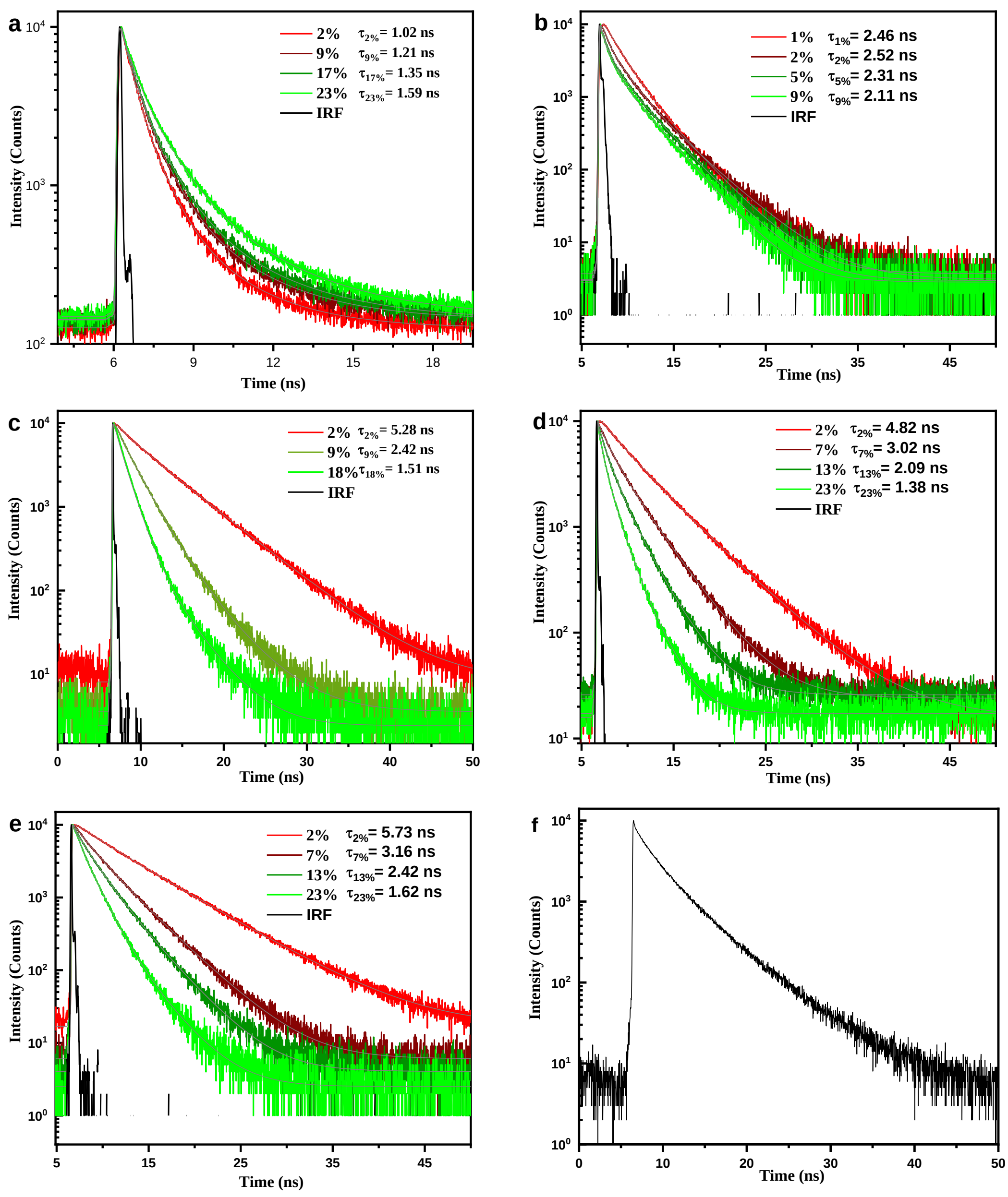

Supplementary Figure 2 Time resolved Photoluminescence of five molecules. Prompt emission decays of 3DPyM-pDTC **(a)**, boron difluoride curcuminoid derivative **(b)**, DABNA-1 **(c)**, DABNA-2 **(d)** and TBN-TPA **(e)** at the in the figures indicated concentrations inside a polystyrene film. Also seen is the prompt emission decay of a neat DABNA-2 film recorded at 77 K **(f)**.

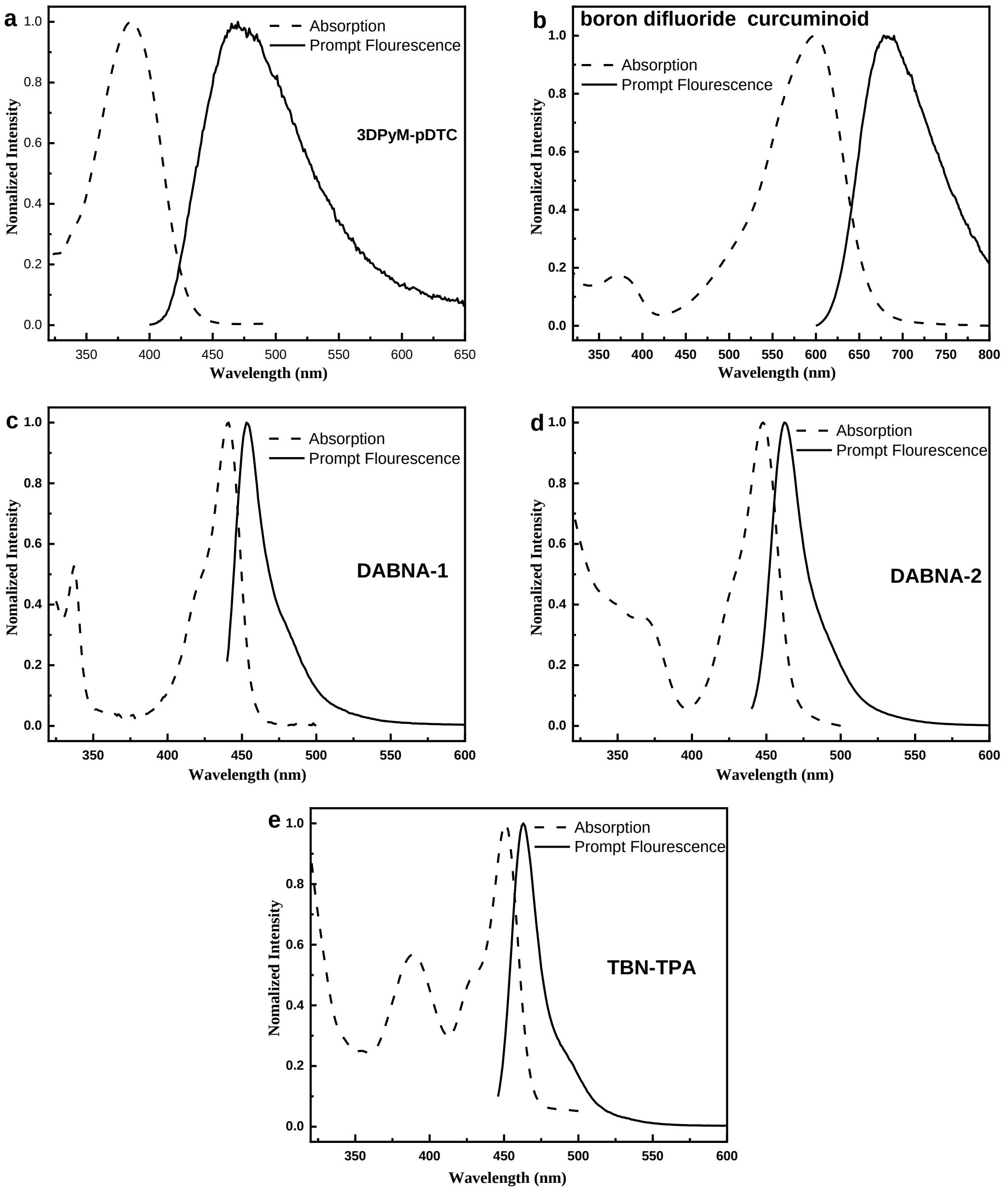

Supplementary Figure 3 Steady state photo luminescence of five molecules. Absorption (dashed black) and prompt emission (solid black) at room temperature of 3DPyM-pDTC **(a)**, boron difluoride curcuminoid derivative **(b)**, DABNA-1 **(c)**, DABNA **(d)** and TBN-TPA **(e)** in polystyrene at low concentration.

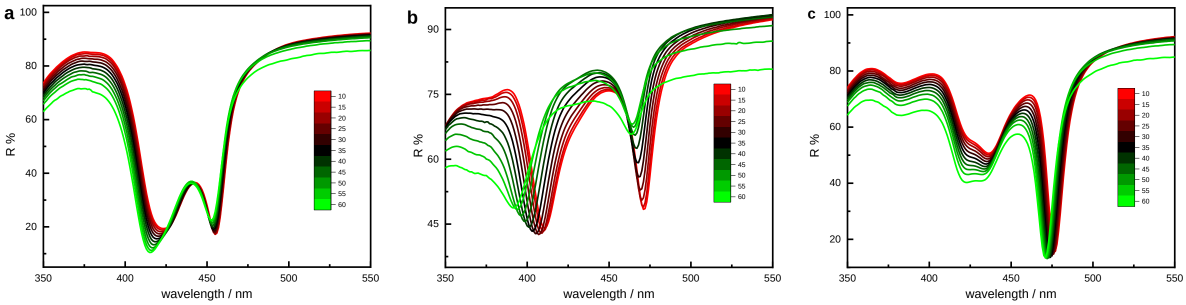

Supplementary Figure 4 Rabi splitting of three molecules inside cavities. Angle dependent reflectance (TE mode) of the cavities containing films of DABNA-1 **(a)**, DABNA-2 **(b)** and TBN-TPA **(c)**.

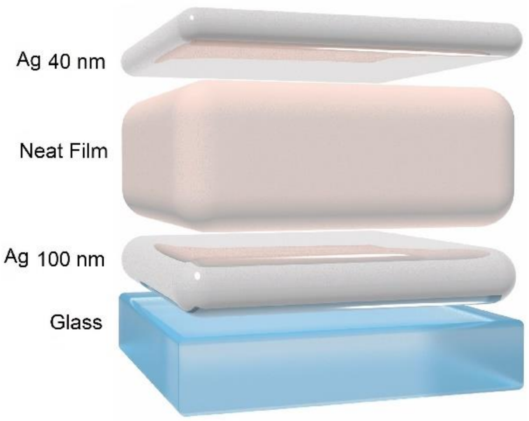

Supplementary Figure 5 The structure of the Fabry-Pérot cavities used in the study.

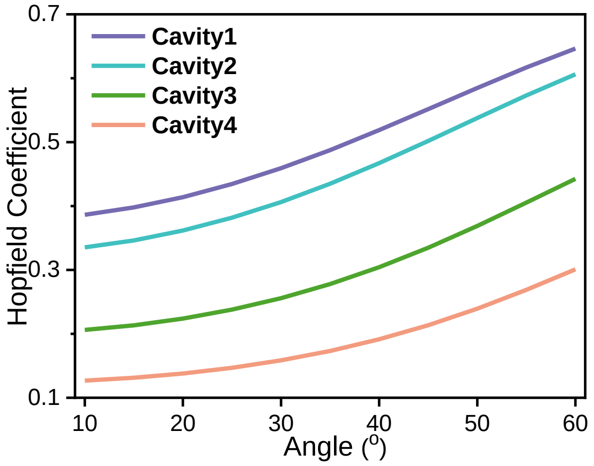

Supplementary Figure 6 Hopfield coefficients for the molecular contribution to P^- in the four cavities.

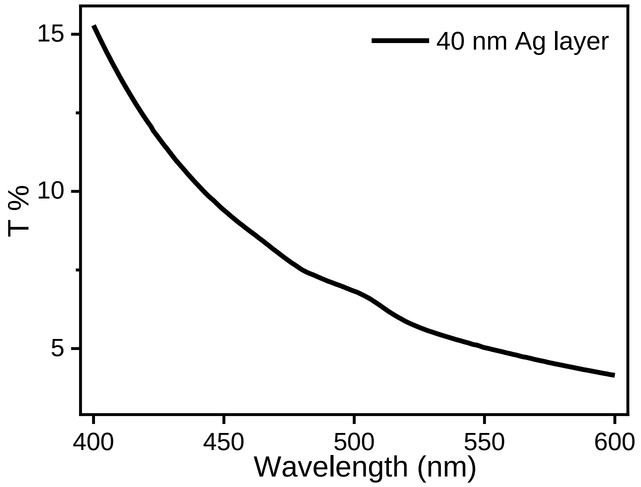

Supplementary Figure 7 Transmittance spectrum for the 40 nm Ag mirror.

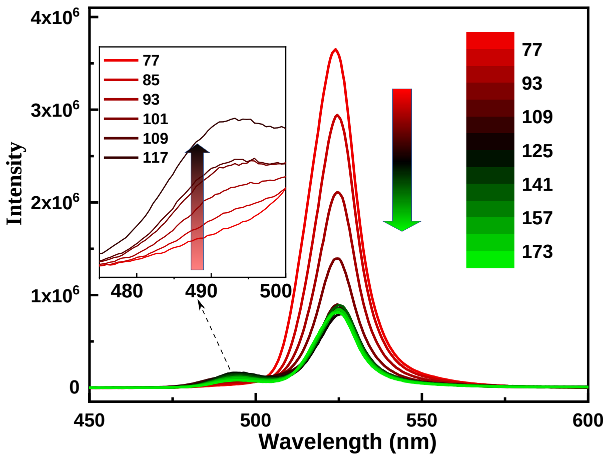

Supplementary Figure 8 Difference between coupled and uncoupled delayed emission. Temperature-dependent (gate delay 100 ns) emission of Cavity3. The insert shows the emission of uncoupled molecules in Cavity3. As the temperature rises, polariton emission diminishes but uncoupled emission is enhanced.

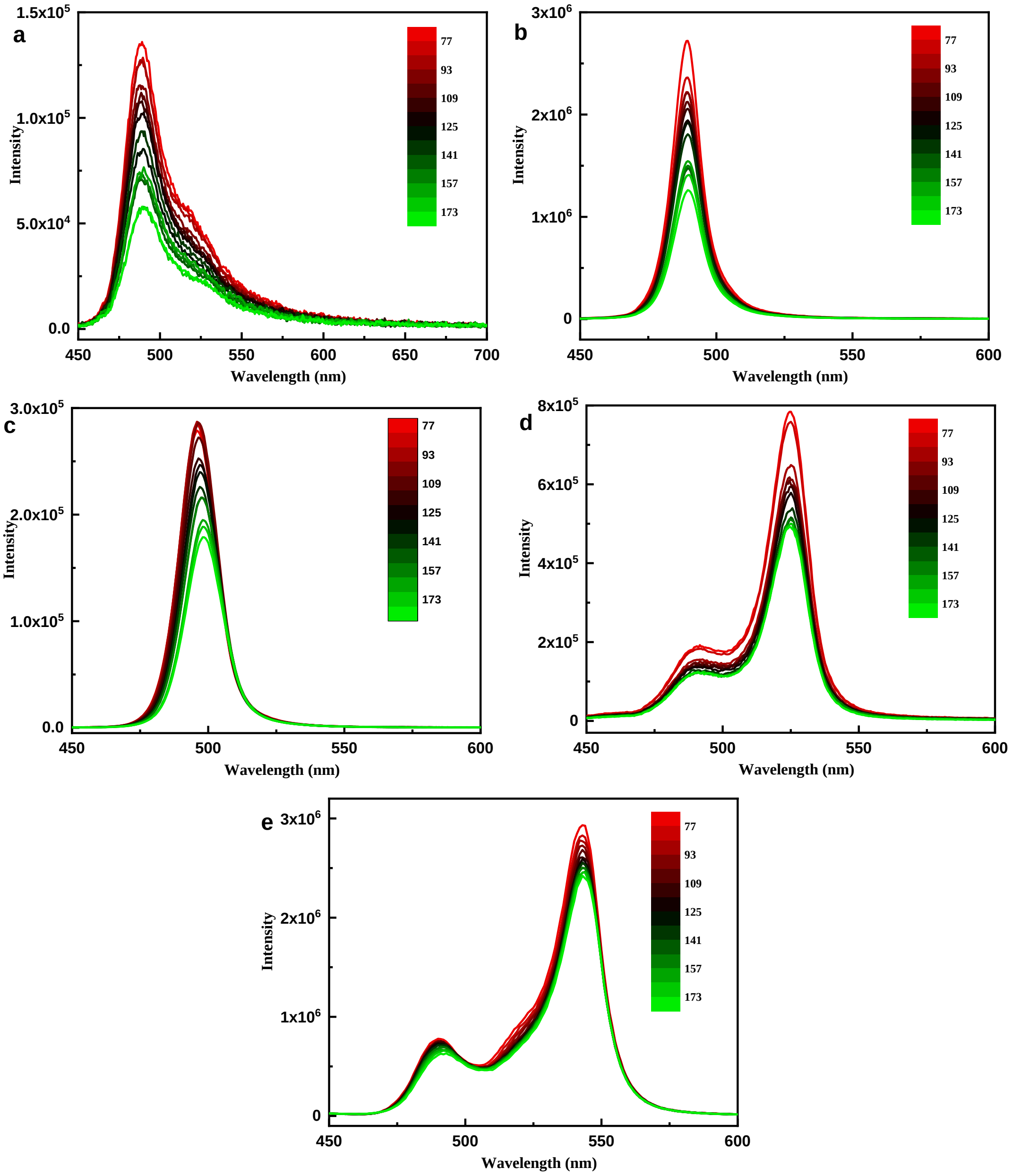

Supplementary Figure 9 Integrated intensity of prompt emission (I_{PF}). Temperature dependent prompt emission of neat film (**a**), Cavity1 (**b**), Cavity2 (**c**) Cavity3 (**d**) and Cavity4 (**e**).

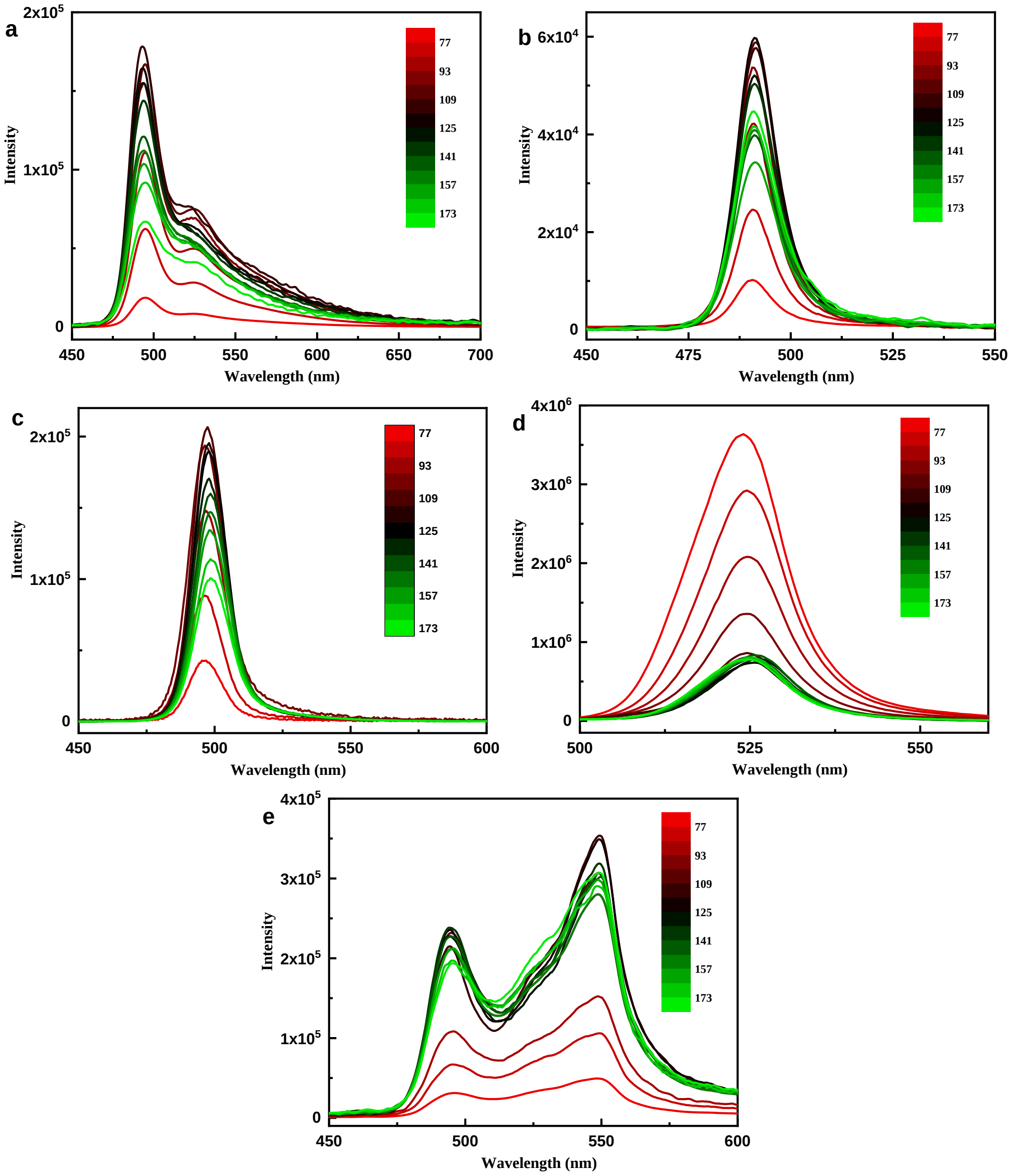

Supplementary Figure 10 Integrated intensity of delayed emission (I_{DF}). Deconvoluted temperature dependent delayed emission of neat film (**a**), Cavity1 (**b**), Cavity2 (**c**) Cavity3 (**d**) and Cavity4 (**e**); In order to get I_{DF} (which should only contain fluorescence and P- emission), shown spectra are deconvoluted with the phosphorescence from a neat film.

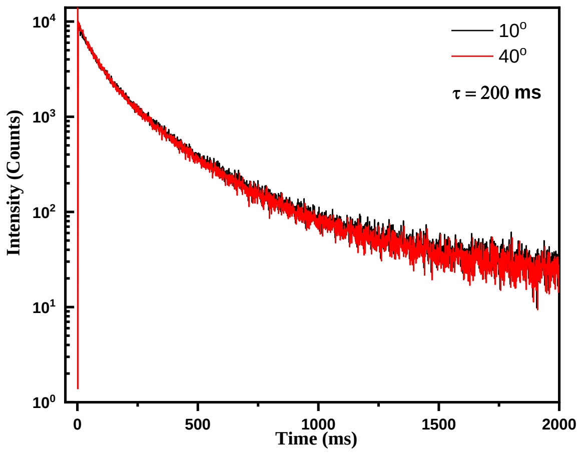

Supplementary Figure 11 Time resolved delayed emission of Cavity4, recorded at 10 and 40 degrees.

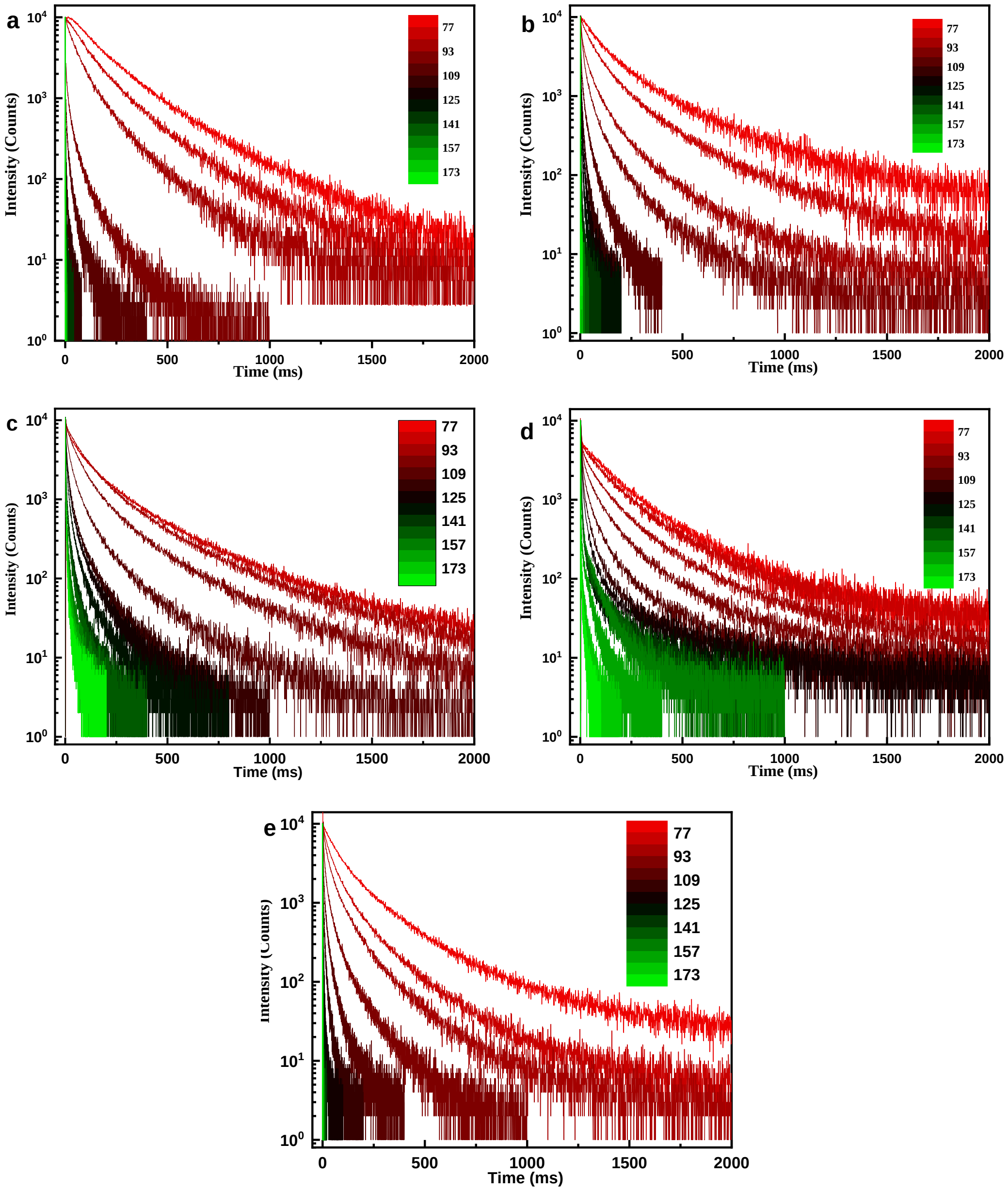

Supplementary Figure 12 Time resolved delayed emission. Temperature dependent delayed emission recorded at the emission maximum of the P^- (or singlet for the neat film) state for neat film (**a**), Cavity1 (**b**), Cavity2 (**c**) Cavity3 (**d**) and Cavity4 (**e**) (gate delay: 3 ms).

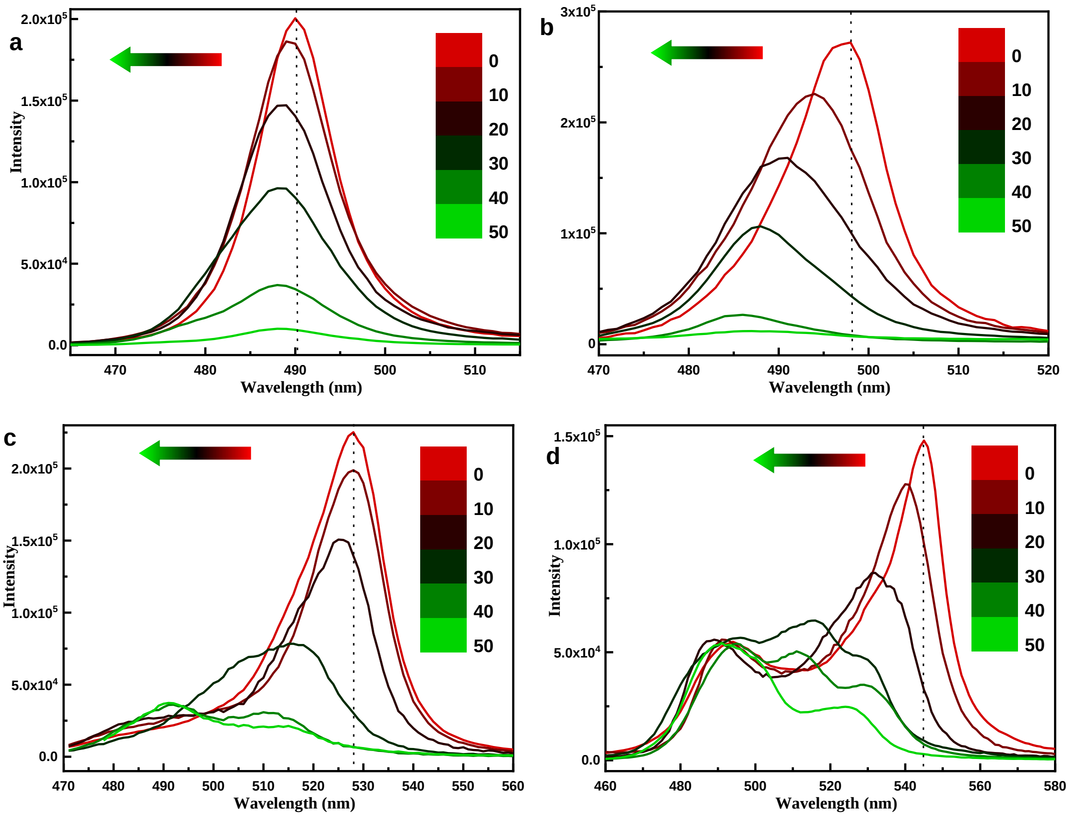

Supplementary Figure 13 Angle dependent emission. Steady state emission of Cavity1 (**a**), Cavity2 (**b**) Cavity3 (**c**) and Cavity4 (**d**) as the angle of the detector is changing from 0 to 50 degree.

3. Supplementary Tables

Supplementary Table 1 The energy levels, Rabi splitting and energy gap of different molecules inside cavities (fitted from the data of Supplementary Figure 4 by the Coupled oscillator model)

	DABNA-1	DABNA-2	TBN-TPA
E_x (eV)	2.834	2.786	2.744
E_c (eV)	2.818	2.793	2.755
$\hbar\Omega_R$ (meV)	247	400	311
ΔE_{ST} (meV)	180	140	140

Supplementary Table 2 The relative rate change in DABNA-2 neat film and Cavities1-4 at different temperatures.

	Neat film		Cavity1		Cavity2		Cavity3		Cavity4	
**T / K**	k_T/k_{77k}	$\ln(k_T/k_{77K})$	k_T/k_{77k}	$\ln(k_T/k_{77K})$	k_T/k_{77k}	$\ln(k_T/k_{77K})$	k_T/k_{77k}	$\ln(k_T/k_{77K})$	k_T/k_{77k}	$\ln(k_T/k_{77K})$
77	1	0	1	0	1	0	1	0	1	0
85	4.69	1.55	3.07	1.12	2.17	0.78	0.98	-0.08	4.89	1.58
93	12.2	2.51	8.43	2.13	3.88	1.36	0.89	-0.11	14.4	2.66
101	43.6	3.77	22.0	3.09	6.69	1.90	0.78	-0.25	50.6	3.92
109	100	4.61	62.8	4.14	12.95	2.56	0.76	-0.28	110	4.71
117	303	5.71	138	4.93	24.06	3.18	0.93	-0.07	152.6	5.03
125	869	6.77	215	5.37	29.64	3.39	1.02	0.02	477	6.17
133	1584	7.37	329	5.79	40.82	3.71	1.22	0.20	1177	7.07
141	2744	7.92	759	6.63	68.92	4.23	1.35	0.30	2201	7.70
149	4950	8.51	2214	7.70	80.61	4.39	1.70	0.53	3671	8.21
157	9147	9.12	3289	8.10	125.72	4.83	2.20	0.79	6757	8.82
165	13449	9.51	3641	8.20	149.64	5.01	4.17	1.43	9376	9.14
173	16076	9.69	5220	8.56	170.12	5.14	12.68	2.54	15595	9.65
181	21853	9.99	7103	8.87	184.39	5.22	23.54	3.16	21530	9.98

Supplementary Table 3 Cavity parameters of Cavity3 and the cavity used in the measurements in Figure 4.

	Cavity3	New Cavity
E_x	2.75	2.75
E_c	2.45	2.47
Detuning	-0.30	-0.32
E_{P-}	2.34	2.36
$\hbar\Omega_R$	0.44	0.42

4. Supplementary References

1 Hatakeyama, T. *et al.* Ultrapure blue thermally activated delayed fluorescence molecules: efficient HOMO–LUMO separation by the multiple resonance effect. *Adv. Mater.* **28**, 2777-2781 (2016).

2 Kim, D.-H. *et al.* High-efficiency electroluminescence and amplified spontaneous emission from a thermally activated delayed fluorescent near-infrared emitter. *Nat. Photonics* **12**, 98-104 (2018).

3 Liang, X. *et al.* Peripheral amplification of multi‐resonance induced thermally activated delayed fluorescence for highly efficient OLEDs. *Angew. Chem. Int. Ed.* **130**, 11486-11490 (2018).

4 Rajamalli, P. *et al.* New molecular design concurrently providing superior pure blue, thermally activated delayed fluorescence and optical out-coupling efficiencies. *J. Am. Chem. Soc.* **139**, 10948-10951 (2017).
